# Prospective Evaluation of the Association Between Arthritis and Cognitive Functions in Middle-Aged and Elderly Chinese

**DOI:** 10.3389/fnagi.2021.687780

**Published:** 2021-10-27

**Authors:** Wenyu Liu, Xue Yang, Xingzhong Jin, Peng Xiu, Ying Wen, Nianwei Wu, Jian Zhao, Dong Zhou, Xiong-Fei Pan

**Affiliations:** ^1^Department of Neurology, West China Hospital, Sichuan University, Chengdu, China; ^2^Department of Epidemiology and Biostatistics, West China School of Public Health and West China Fourth Hospital, Sichuan University, Chengdu, China; ^3^Centre for Big Data Research in Health, University of New South Wales, Sydney, NSW, Australia; ^4^Department of Orthopedics, West China Hospital, Sichuan University, Chengdu, China; ^5^Department of Communicable Diseases Control and Prevention, Shenzhen Center for Disease Control and Prevention, Shenzhen, China; ^6^Population Health Sciences, Bristol Medical School, University of Bristol, Bristol, United Kingdom; ^7^MRC Integrative Epidemiology Unit, University of Bristol, Bristol, United Kingdom; ^8^Ministry of Education Key Laboratory of Environment and Health and State Environmental Protection Key Laboratory of Environment and Health, Department of Epidemiology and Biostatistics, School of Public Health, Tongji Medical College, Huazhong University of Science and Technology, Wuhan, China; ^9^Division of Epidemiology, Department of Medicine, Vanderbilt University Medical Center, Nashville, TN, United States; ^10^Faculty of Medicine, The George Institute for Global Health, University of New South Wales, Sydney, NSW, Australia

**Keywords:** arthritis, cognitive functions, mental status, episodic memory, Chinese

## Abstract

**Background:** Assessing the relation between arthritis and cognitive impairment could expand the understanding of health consequences of arthritis. The aim was to prospectively examine the association between arthritis and cognitive functions among middle-aged and elderly Chinese.

**Methods:** Our analyses were based on data from the nationwide China Health and Retirement Longitudinal Study (2011–2016). Arthritis was ascertained by self-reported doctor diagnosis during the baseline survey. Cognitive functions were evaluated in three domains including episodic memory, mental status, and global cognition. Linear mixed models were employed to assess the association between baseline arthritis and cognition functions.

**Results:** Of 7,529 Chinese adults, 49.79% were men, and mean age was 57.53 years. During a follow-up of 4 years, participants with baseline arthritis showed lower scores of episodic memory [β = −0.08; 95% confidence interval (CI): −0.14, −0.03], mental status (β = −0.14; 95% CI: −0.22, −0.05), and global cognition (β = −0.22; 95% CI: −0.34, −0.11), compared to those without arthritis. In addition, participants with arthritis showed increased rates of decline in mental status and global cognition by 0.04 (95% CI: 0.01, 0.08) and 0.05 (95% CI: 0.01, 0.09) units per year, respectively.

**Conclusion:** Arthritis was associated with subsequent risk of poorer cognitive functions and slightly faster declines in cognitive functions among Chinese middle-aged and elderly adults. Our findings should be confirmed in future large prospective studies in Chinese and other populations.

## Introduction

Cognitive impairment is a common aging-related chronic condition that can progress as a preclinical stage to dementia (Arvanitakis et al., 2019). Globally, mild cognitive impairment is prevalent in over 10% of people aged 70 years or older (Petersen R. C. et al., 2018). In China, the prevalence of mild cognitive impairment could reach up to 15% in people aged 60 years or older (Xue et al., 2018), and the prevalence of dementia was 5.6% in people aged 65 years or older (Huang Y. et al., 2019). Thus, cognitive impairment is emerging as a major population health challenge both in China and worldwide, and investigations for its risk factors may inform the strategies to combat the challenge.

Arthritis is a group of chronic joint diseases characterized by pain and stiffness in one or more joints, of which osteoarthritis and rheumatoid arthritis are the two most common types. While physical disability is frequently noted for arthritis, health effects of arthritis can extend beyond the musculoskeletal system, such as psychiatric and cognitive disorders (Sturgeon et al., 2016; Ungprasert et al., 2016; Xue et al., 2020). Evidence is accumulated for a high prevalence of cognitive impairment in patients with rheumatoid arthritis (Meade et al., 2018). However, the findings for the association between arthritis and cognitive functions are still mixed. Midlife rheumatoid arthritis was associated with higher risk of cognitive impairment in later life in Finland (Wallin et al., 2012), while the severity of rheumatoid arthritis was positively associated with risk of cognitive impairment in Thailand (Katchamart et al., 2019). On the contrary, another prospective study in Mexico did not find evidence of higher risk of cognitive impairment or dementia associated with arthritis (Veeranki et al., 2017). Of note, all these studies were conducted in non-Chinese populations, and their sample sizes were generally small. Since arthritis could afflict over 20% of people aged over 50 years in China (Brennan-Olsen et al., 2017), assessing the relation between arthritis and cognitive impairment could add to the extant body of evidence.

In this context, we utilized data from the China Health and Retirement Longitudinal Study (CHARLS) to examine the associations of arthritis with cognitive functions as well as rates of cognitive decline in domains of episodic memory, mental status, and global cognition. We also assessed the heterogeneity of the association with cognitive functions in different subpopulations. While the CHARLS data have been utilized to explore the prevalence and risk factors of arthritis in prior analyses (Li et al., 2015), our current work could expand the understanding of its health consequences.

## Materials and Methods

### Population

The CHARLS is an ongoing nationwide longitudinal study that aimed to explore aging-related issues in China. The detailed study design, methodology, and procedures of the CHARLS were reported elsewhere (Zhao et al., 2014). Briefly, the CHARLS enrolled a total of 17,708 adults aged 45 years or older via a multi-stage probability sampling procedure from 450 communities of 28 provinces in China in 2011–2012 (baseline). Using computer-assisted standardized questionnaires, trained staff collected information in face-to-face interviews on demographic and socioeconomic factors, lifestyles and behaviors, and health status and functioning. In addition, blood samples were collected at baseline for biomarker measurements. Two follow-ups in 2013–2014 and 2015–2016 were completed according to similar procedures at baseline, and response rates were 88.30 and 87.15%, respectively. The CHARLS was approved by the Biomedical Ethics Review Committee of Peking University (IRB00001052-11015) and informed consent was obtained from all participants.

In our analyses, we excluded study participants who (1) reported memory-related disease at baseline such as Alzheimer’s disease, brain atrophy and Parkinson’s disease, or had data missing for memory-related disease (*n* = 490); (2) had data missing for arthritis at baseline (*n* = 31); (3) had data missing for cognitive functions at baseline or at either of the two follow-ups (*n* = 8,191); or (4) had data missing for major sociodemographics, and lifestyle and behavior covariates (*n* = 1,467) including sex (*n* = 8), residential area (*n* = 8), education (*n* = 3), smoking status (*n* = 1), BMI (*n* = 1,347), and comorbidities (*n* = 100). The final study population included a total of 7,529 participants for primary analyses (Supplementary Figure 1).

### Ascertainment of Baseline Arthritis

Baseline arthritis was self-reported doctor diagnosis at baseline. History of physician-diagnosed arthritis was inquired by trained staff using the question “*Have you been diagnosed with arthritis or rheumatism by a doctor*?” If participants answered “yes” to the question, they were regarded as having arthritis at baseline.

### Measurement of Cognitive Functions

Cognitive functions were assessed at baseline and two follow-up visits using two composite measurements covering episodic memory and mental status (Yang et al., 2020).

(1) Episodic memory: Immediate memory and delayed memory were tested in this component. Trained staff read 10 Chinese words in succession to the participants, and required them to repeat the words in any order immediately (immediate memory), and to recall the words 5 min later (delayed memory) (Crimmins et al., 2011; Bender et al., 2017). The scores of immediate memory and delayed memory were calculated based on the number of words that were correctly recalled. The overall score was computed as the average of these two parts, with a range from 0 to 10.
(2) Mental status: The component comprised time orientation, numerical ability and picture drawing. The time orientation test required participants to recall today’s date (year, month, and day), the day of the week, and the current season. The numerical ability test required participants to do a serial subtraction of 7 starting from 100 (up to 5 times). The score of numerical ability was the number of correct answers, but would be reduced by half if an aid like a pen or paper was used. The picture drawing test measured the ability to draw a picture of two overlapping pentagons. Participants who successfully reproduced the picture received 1 point, and those who failed to do so received a score of 0 (Sha et al., 2018). The total score of mental status ranged from 0 to 11.

We assessed the global cognition of participants by summing up the scores of episodic memory and mental status. The total score ranged from 0 to 21, and a higher score indicated a higher level of cognitive function.

### Covariate Measurements

At baseline, participant characteristics including year of birth, sex, marital status, residential area, lifestyle (e.g., smoking and drinking status), and comorbidities (i.e., hypertension, diabetes mellitus, stroke, heart disease, lung disease, and cancer) were collected. Marital status was recorded as married or unmarried. Education level was categorized as illiterate, primary/middle school, and senior high school or above. Residential area included rural and urban areas. BMI was calculated as weight divided by the square of height (kg/m^2^). The following Chinese criteria were used to categorize participants by BMI: < 18.5 kg/m^2^ for underweight, 18.5–23.9 kg/m^2^ for normal weight, 24.0–27.9 kg/m^2^ for overweight, and 28.0 kg/m^2^ or higher for obesity (Pan et al., 2021). Smoking and drinking status was categorized into three groups: never, former, and current. Diabetes mellitus was defined by self-reported doctor diagnosis or glucose measures (fasting blood glucose ≥ 126 mg/dL or glycosylated hemoglobin level ≥ 6.5%) (American Diabetes Association [ADA], 2020). Baseline hypertension was determined by self-reported doctor diagnosis or blood pressure (systolic blood pressure ≥ 140 mmHg or diastolic blood pressure ≥ 90 mmHg) (Liu, 2011). Other comorbidities (i.e., stroke, heart disease, lung disease, and cancer) were ascertained by self-reported doctor diagnosis. The number of baseline comorbidities was categorized into four groups: none, one, two, and three or more.

### Statistic Analysis

Continuous variables including age and BMI were summarized as mean and standard deviation, and categorical variables were presented as frequency and percentage. Student’s *t*-test and Pearson’s chi-square test were used to compare characteristics between participants with and without baseline arthritis.

Linear mixed models were applied to estimate the associations between arthritis and cognitive functions after sequential adjustments for years since baseline, age, sex, marital status, education level, residential area, BMI, smoking status, drinking status, and the number of comorbidities in three models. We conducted subgroup analyses by sex, age, and BMI to determine whether the associations differed between subgroups. In a sensitivity analysis, we performed multiple imputations for 1,467 participants with missing data of covariates including age, sex, residential area, education level, BMI, smoking status, drinking status, marital status, and number of comorbidities at baseline. Assuming that data in our study were randomly missing, we replaced missing data with imputed values obtained from five duplicate datasets, which were generated to minimize the sampling variability, and the final effect estimates were calculated by combining the effect estimates from the five imputed datasets.

In order to evaluate the relationship between arthritis at baseline and the rate of cognitive decline, we tested the statistical significance of the interaction term of baseline arthritis and years since baseline survey in the final linear mixed model after adjusting for potential confounders mentioned above. Stata 15.0 (StataCorp LLC, College Station, Texas, United States) was used to perform all analyses in this study, and statistical significance was defined as two-tailed *P* < 0.05.

## Results

### Baseline Characteristics

Of 7,529 study participants, 49.79% were men and mean age was 57.53 years (standard deviation, 8.85). The prevalence of arthritis was 32.22% at baseline. Compared with participants without arthritis, participants who had arthritis at baseline were more likely to be women, from rural areas, poorly educated, never smokers, or drinkers, and with a larger number of comorbidities (Table 1). We did not find statistically significant differences in most baseline characteristics between the included 7,529 participants and 1,467 participants who were excluded due to data missing for major covariates (Supplementary Table 1).

**TABLE 1:** Baseline characteristics of study participants in the CHARLS.

Characteristics	Total (%)	Arthritis		*P*-values
		Yes	No	
Total	7,529 (100.00)	2,426 (32.22)	5,103 (67.78)	
Age, mean (SD), years	57.53 (8.85)	58.28 (8.45)	57.17 (9.02)	<0.001
BMI, mean (SD), kg/m^2^	23.81 (3.71)	24.00 (3.78)	23.72 (3.67)	0.002
Sex, n (%)				<0.001
Male	3,749 (49.79)	1,030 (42.46)	2,719 (53.28)	
Female	3,780 (50.21)	1,396 (57.54)	2,384 (46.72)	
Residence, n (%)				<0.001
Rural	5,744 (76.29)	1,926 (79.39)	3,818 (74.82)	
Urban	1,785 (23.71)	500 (20.61)	1,285 (25.18)	
Marital status, n (%)				0.107
Married	7,482 (99.38)	2,416 (99.59)	5,066 (99.27)	
Unmarried	47 (0.62)	10 (0.41)	37 (0.73)	
Education level, n (%)				<0.001
Illiterate	1,325 (17.60)	486 (20.03)	839 (16.44)	
Primary/middle school	3,198 (42.48)	1,141 (47.03)	2,057 (40.31)	
High school or above	3,006 (39.93)	799 (32.93)	2,207 (43.25)	
Smoking status, n (%)				<0.001
Never smoking	4,476 (59.45)	1,541 (63.52)	2,935 (57.52)	
Former smoking	669 (8.89)	203 (8.37)	466 (9.13)	
Current smoking	2,384 (31.66)	682 (28.11)	1,702 (33.35)	
Drinking status, n (%)				0.005
Never drinking	5,105 (67.80)	1,685 (69.46)	3,420 (67.02)	
Former drinking	432 (5.74)	154 (6.35)	278 (5.45)	
Current drinking	1,992 (26.46)	587 (24.20)	1,405 (27.53)	
Comorbidities, n (%)				<0.001
None	3,473 (46.13)	968 (39.90)	2,505 (49.09)	
One	2,780 (36.92)	918 (37.84)	1,862 (36.49)	
Two	988 (13.12)	396 (16.32)	592 (11.60)	
Three or more	288 (3.83)	144 (5.94)	144 (2.82)	

*BMI, body mass index; CHARLS, China Health and Retirement Longitudinal Study; SD, standard deviation.*

### Baseline Arthritis and Subsequent Cognitive Functions

During the 4-year follow-up, participants with arthritis at baseline had lower scores in episodic memory (β = −0.08; 95% CI: −0.14, −0.03), mental status (β = −0.14; −0.22, −0.05), and global cognition (β = −0.22; −0.34, −0.11) than those without arthritis (Table 2). With multiple imputations for data missing, the inverse associations did not change appreciably for episodic memory (β = −0.08; 95% CI: −0.12, −0.04), mental status (β = −0.17; 95% CI: −0.23, −0.10), and global cognition (β = −0.25; 95% CI: −0.34, −0.17) (Supplementary Table 2). We did not observe heterogeneities in the associations between arthritis and cognitive functions across sex, age, and BMI groups (*P* for interaction ≥ 0.141 for all; Table 3). Despite no statistical significance, the inverse associations seemed stronger in men and those with obesity with respect to episodic memory, mental status, and global cognition.

**TABLE 2:** Associations between baseline arthritis and subsequent cognitive functions.

	Model 1: β (95% CI)^a^	Model 2: β (95% CI)^a^	Model 3: β (95% CI)^a^
Episodic memory	−0.22 (−0.29, −0.16)	−0.09 (−0.15, −0.04)	−0.08 (−0.14, −0.03)
Mental status	−0.40 (−0.51, −0.30)	−0.14 (−0.23, −0.05)	−0.14 (−0.22, −0.05)
Global cognition	−0.63 (−0.77, −0.48)	−0.24 (−0.35, −0.12)	−0.22 (−0.34, −0.11)

*CI, confidence interval.*

*Model 1: adjusted for years since baseline (0, 2, and 4 years).*

*Model 2: adjusted for age (continuous, years), sex (male and female), marital status (married and unmarried), education (illiterate, primary/middle school, and high school or above), residential area (rural and urban), BMI (continuous, kg/m^2^), smoking status (never, former, and current), drinking status (never, former, and current), and variables in Model 1.*

*Model 3: adjusted for comorbidities (none, one, two, and three or more) and variables in Model 2.*

*^*a*^For arthritis vs. no arthritis.*

**TABLE 3:** Associations between baseline arthritis and subsequent cognitive functions in subgroups.

Subgroups	Number of	Episode memory		Mental status		Global cognition	
	participants						
		β (95% CI)	*P* for interaction	β (95% CI)	*P* for interaction	β (95% CI)	*P* for interaction
Sex			0.412		0.877		0.816
Male	3,749	−0.11 (−0.19, −0.03)		−0.16 (−0.28, −0.04)		−0.27 (−0.44, −0.11)	
Female	3,780	−0.06 (−0.13, −0.02)		−0.13 (−0.25, −0.001)		−0.19 (−0.36, −0.02)	
Age			0.303		0.256		0.141
45–50	1,821	−0.11 (−0.23, −0.01)		−0.16 (−0.33, −0.02)		−0.28 (−0.53, −0.04)	
51–60	2,773	−0.08 (−0.18, −0.01)		−0.23 (−0.38, −0.09)		−0.33 (−0.52, −0.13)	
>60	2,935	−0.09 (−0.18, −0.01)		−0.08 (−0.22, 0.06)		−0.16 (−0.35, 0.03)	
BMI^*a*^			0.318		0.435		0.963
Underweight	365	−0.11 (−0.39, 0.17)		−0.08 (−0.53, 0.38)		−0.21 (−0.84, 0.41)	
Normal	3,850	−0.05 (−0.13, 0.03)		−0.17 (−0.30, −0.05)		−0.22 (−0.39, −0.05)	
Overweight	2,378	−0.09 (−0.19, 0.01)		−0.08 (−0.23, 0.07)		−0.18 (−0.38, 0.03)	
Obese	936	−0.18 (−0.34, −0.03)		−0.21 (−0.44, 0.02)		−0.41 (−0.72, −0.09)	

*BMI, body mass index; CI, confidence interval.*

*Adjusted for years since baseline (0, 2, and 4 years), age (continuous, years), sex (male and female), marital status (married and unmarried), education (illiterate, primary/middle school, and high school or above), residential area (rural and urban), time (continuous, year), BMI (continuous, kg/m^2^), smoking status (never, former, and current), drinking status (never, former, and current), and comorbidities (none, one, two, and three or more).*

*^*a*^The following Chinese criteria were used to categorize participants by BMI: < 18.5 kg/m^2^ for underweight, 18.5–23.9 kg/m^2^ for normal weight, 24.0–27.9 kg/m^2^ for overweight, and 28.0 kg/m^2^ or higher for obesity.*

Cognitive functions declined in episodic memory, mental status, and global cognition during the 4-year follow-up (*P* < 0.001 for all; Table 4). As indicated by the interaction between baseline arthritis and years since baseline, participants with baseline arthritis showed faster decline in mental status (*P* for interaction = 0.010) and global cognition (*P* for interaction = 0.027) (Table 4). Rates of decline in mental status and global cognition increased by 0.04 (β = 0.04; 95% CI: 0.01, 0.08) and 0.05 (β = 0.05; 95% CI: 0.01, 0.09) units per year in participants with arthritis compared with those without arthritis. However, there was a lack of evidence for faster decline in episodic memory for participants with arthritis than those without (*P* for interaction = 0.849).

**TABLE 4:** Changes of cognitive functions by baseline arthritis.

	Intercept		Years since baseline		Arthritis × years since baseline	
	β (95% CI)	*P*-value	β (95% CI)	*P*-value	β (95% CI)	*P*-value
Episodic memory	4.64 (4.34, 4.95)	<0.001	−0.07 (−0.08, −0.05)	<0.001	0.002 (−0.02, 0.02)	0.849
Mental status	6.42 (5.95, 6.89)	<0.001	−0.06 (−0.08, −0.04)	<0.001	0.04 (0.01, 0.08)	0.010
Global cognition	11.04 (10.39, 11.68)	<0.001	−0.12 (−0.14, −0.09)	<0.001	0.05 (0.01, 0.09)	0.027

*CI, confidence interval.*

*Adjusted for years since baseline (0, 2, and 4 years), age (continuous, years), sex (male and female), marital status (married and unmarried), education (illiterate, primary/middle school, and high school or above), residential area (rural and urban), time (continuous, year), BMI (continuous, kg/m^2^), smoking status (never, former, and current), drinking status (never, former, and current), and comorbidities (none, one, two, and three or more).*

## Discussion

In this large nationwide longitudinal study among ∼ 7,500 Chinese adults aged 45 years or older, we documented inverse associations between arthritis and cognitive functions in a 4-year follow-up. Participants with arthritis had poorer cognitive functions than those without arthritis, as reflected in lower scores of episodic memory, mental status, and global cognition. In addition, there was slightly faster decline in cognitive functions in those with arthritis than those without.

The national prevalence of self-reported arthritis was up to 32.2% in adults aged 45 years or above in our study, which was equivalent to the self-reported prevalence of 22–30% in Chinese aged 50 years or older from a World Health Organization (WHO) collaborative study (Brennan-Olsen et al., 2017). The slight difference could be attributable to the fact that the WHO study only enrolled participants from 8 provinces of China, most of which were located in more affluent eastern China, while our study sample almost covered all regions of mainland China (28 provinces). Our estimated prevalence of arthritis in China almost reached a level observed in developed countries such as the US (Helmick et al., 2008). The high prevalence of arthritis in middle-aged and elderly Chinese contextualized within a broad health impact framework our research question on its association with cognitive impairment.

To the best of our knowledge, our study was the first to prospectively examine the relations of arthritis and cognitive functions using a national sample in mainland China. The poorer cognitive functions associated with arthritis observed in China was consistent with the evidence of cognitive impairment in adults with rheumatoid arthritis from a meta-analysis of 15 studies (Meade et al., 2018). However, all the included studies in this meta-analysis were cross-sectional, so no temporal association could be determined. Four major prospective studies have examined the associations between arthritis and cognitive impairment in different countries (Wallin et al., 2012; Baker et al., 2017; Veeranki et al., 2017; Katchamart et al., 2019), but there was a lack of consensus in their findings. Consistent with our finding, a prospective study among 1,449 Finish participants with a mean age of ∼50 years found that midlife arthritis as a whole was associated with higher risk of cognitive impairment, dementia, and Alzheimer’s disease in late life during a follow-up of 21 years (Wallin et al., 2012). Similarly, in 464 patients (mean age, 59.2 years) with rheumatoid arthritis in Thailand, higher rheumatoid arthritis disease activity correlated with higher risk of cognitive impairment during a median follow-up of 5.2 years (Katchamart et al., 2019). However, in a study among 2,681 Mexicans aged 60 years or older (mean age, 66.5 years), arthritis as a whole was associated with functional impairment but not cognitive impairment or dementia after 11 years of follow-up (Veeranki et al., 2017). Furthermore, in prospective analyses of data from the Health and Retirement Study in the US, arthritis was associated with neither cognitive impairment nor dementia in 9,728 participants aged 65 years or older during a 6-year follow-up (Baker et al., 2017). The same study also showed that participants developed cognitive impairment at a similar rate to those without arthritis, which was inconsistent with the observed higher rate of decline in cognitive functions in our study among Chinese. These studies from different countries were characterized by different sample sizes, durations of follow-ups, age distributions, definitions of cognitive functions, and even background participant characteristics such as lifestyles and risk behaviors. In particular, participants seemed younger in the former two studies (Wallin et al., 2012; Katchamart et al., 2019) and ours that reported higher risk of cognitive impairment associated with arthritis.

Cohort studies that used dementia as the primary endpoint instead of cognitive impairment also addressed similar research questions. Two large cohort studies showed that osteoarthritis was associated with higher risk of dementia (Huang et al., 2015; Innes and Sambamoorthi, 2020), which reinforces our finding solely for cognitive functions. A population-wide study with a 4-year follow-up in Chinese Taiwan showed participants with osteoarthritis (*n* = 35,149) was 1.25 times as likely to develop dementia as participants without osteoarthritis (*n* = 70,298) (Huang et al., 2015), while another US study with a 2-year follow-up among 16,934 community-dwelling participants aged 65 years or older showed similarly higher risk of dementia for participants with osteoarthritis, particularly those with both osteoarthritis and pain (Innes and Sambamoorthi, 2020). In contrast to osteoarthritis, results for rheumatoid arthritis were less consistent: a nested case-control in Korea did not find evidence for the association of rheumatoid arthritis with dementia (Min et al., 2020), while a population-wide cohort study in Chinese Taiwan showed a positive association for autoimmune rheumatic diseases (Lin et al., 2018). In addition, recent Mendelian randomization analyses also reported conflicting findings regarding the causal association between rheumatoid arthritis and Alzheimer’s disease (Policicchio et al., 2017; Bae and Lee, 2019). The inconsistent results between osteoarthritis and rheumatoid arthritis and even within rheumatoid arthritis may suggest that the types of arthritis could have differential effects and that unascertained confounding and bias could exist in different studies, so well-designed large prospective studies are still required in future.

The mechanisms underlying the link between arthritis and cognitive disorders are largely unclear. Potential mechanisms may involve chronic inflammation (Al-Khazraji et al., 2018), immune changes (Broce et al., 2018; Petersen L.E. et al., 2018), and persistent pain and fatigue (Bushnell et al., 2013). However, mechanistic studies are still insufficient to confirm any of these mechanisms, and should be scientifically designed and implemented in future. Despite uncertainties around the link between arthritis and cognitive impairment, precautionary measures such as routine screening for cognitive functions may be advised in clinical practice for patients with arthritis.

While our study has advantages of prospective study nature, large sample size, and collection of repeated measures, we acknowledge that certain limitations should be addressed in future work. First, we could not differentiate the different types of arthritis in our analyses due to data limitations, and their differential associations with cognitive functions should be explored prospectively in future. Second, a considerable number of participants with data missing on major covariates were excluded from analyses, and such exclusion might induce selection bias inherent in our findings. However, our sensitivity analysis using multiple imputations for data missing generally found similar results to those from the main analyses. Third, we did not have information for arthritis medications in our analyses, and thus could not rule out their potential confounding effect in the link between arthritis and cognitive impairment. For example, anti-rheumatic drugs appeared to reduce risk of dementia in patients with rheumatoid arthritis (Huang L. C. et al., 2019; Newby et al., 2020). Future studies on this topic should collect information for anti-inflammatory drugs and adjust for their confounding effects in statistical analyses. Fourth, given the chronicity of arthritis and cognitive declines, the 4-year follow-up might be short to examine the causal correlation between arthritis and cognitive functions, and reverse causality could not be avoided. Future studies with long-term follow-ups should be used to explore similar topics, with a focus on the dementia outcome.

## Conclusion

In conclusion, participants with baseline arthritis showed poorer cognitive functions and slightly faster decline in cognitive functions than participants without arthritis. While these findings need to be validated in other longitudinal studies in China, they highlight a need for monitoring cognitive functions among middle-aged and elderly Chinese patients with arthritis.

## Data Availability Statement

Publicly available datasets were analyzed in this study. The data can be found here: https://opendata.pku.edu.cn/dataverse/CHARLS.

## Ethics Statement

The CHARLS was approved by the Biomedical Ethics Review Committee of Peking University (IRB00001052-11015), and informed consent was obtained from all participants.

## Author Contributions

WL: study design, data analysis, and manuscript writing. XY: methodology and data analysis. XJ, PX, NW, and JZ: data interpretation and manuscript revisions. DZ: study design and manuscript revisions. X-FP: study design, data interpretation, and manuscript revisions. All authors contributed to the article and approved the submitted version.

## Conflict of Interest

The authors declare that the research was conducted in the absence of any commercial or financial relationships that could be construed as a potential conflict of interest.

## Publisher’s Note

All claims expressed in this article are solely those of the authors and do not necessarily represent those of their affiliated organizations, or those of the publisher, the editors and the reviewers. Any product that may be evaluated in this article, or claim that may be made by its manufacturer, is not guaranteed or endorsed by the publisher.

## References

[B1] Al-Khazraji B. K. Appleton C. T. Beier F. Birmingham T. B. Shoemaker J. K. (2018). Osteoarthritis, cerebrovascular dysfunction and the common denominator of inflammation: a narrative review. *Osteoarthritis Cartilage* 26 462–470. 10.1016/j.joca.2018.01.011 29406252

[B2] American Diabetes Association [ADA] (2020). Classification and diagnosis of diabetes: standards of medical care in diabetes. *Diabetes Care* 43 S14–S31. 10.2337/dc20-S002 31862745

[B3] Arvanitakis Z. Shah R. C. Bennett D. A. (2019). Diagnosis and management of dementia: review. *JAMA* 322 1589–1599. 10.1001/jama.2019.4782 31638686 PMC7462122

[B4] Bae S. C. Lee Y. H. (2019). Causal association between rheumatoid arthritis and a decreased risk of Alzheimer’s disease : a mendelian randomization study. *Z. Rheumatol.* 78 359–364. 10.1007/s00393-018-0504-8 29974225

[B5] Baker N. A. Barbour K. E. Helmick C. G. Zack M. Al Snih S. (2017). Arthritis and cognitive impairment in older adults. *Rheumatol. Int.* 37 955–961. 10.1007/s00296-017-3698-1 28337526 PMC5543988

[B6] Bender A. C. Austin A. M. Grodstein F. Bynum J. P. W. (2017). Executive function, episodic memory, and medicare expenditures. *Alzheimer’s Dementia* 13 792–800. 10.1016/j.jalz.2016.12.013 28174070 PMC5496776

[B7] Brennan-Olsen S. L. Cook S. Leech M. T. Bowe S. J. Kowal P. Naidoo N. (2017). Prevalence of arthritis according to age, sex and socioeconomic status in six low and middle income countries: analysis of data from the World Health Organization study on global AGEing and adult health (SAGE) Wave 1. *BMC Musculoskelet Disord.* 18:271. 10.1186/s12891-017-1624-z 28633661 PMC5479046

[B8] Broce I. Karch C. M. Wen N. Fan C. C. Mayhaus M. (2018). Immune-related genetic enrichment in frontotemporal dementia: an analysis of genome-wide association studies. *PLoS Med.* 15:e1002487. 10.1371/journal.pmed.1002487 29315334 PMC5760014

[B9] Bushnell M. C. Ceko M. Low L. A. (2013). Cognitive and emotional control of pain and its disruption in chronic pain. *Nat. Rev. Neurosci.* 14 502–511. 10.1038/nrn3516 23719569 PMC4465351

[B10] Crimmins E. M. Kim J. K. Langa K. M. Weir D. R. (2011). Assessment of cognition using surveys and neuropsychological assessment: the health and retirement study and the aging, demographics, and memory study. *J. Gerontol. Series B Psychol. Sci. Soc. Sci.* 66 i162–i171. 10.1093/geronb/gbr048 21743047 PMC3165454

[B11] Helmick C. G. Felson D. T. Lawrence R. C. Gabriel S. Hirsch R. Kwoh C. K. (2008). Estimates of the prevalence of arthritis and other rheumatic conditions in the United States. Part I. *Arthritis Rheum* 58 15–25. 10.1002/art.23177 18163481

[B12] Huang L. C. Chang Y. H. Yang Y. H. (2019). Can disease-modifying anti-rheumatic drugs reduce the risk of developing dementia in patients with rheumatoid arthritis? *Neurotherapeutics* 16 703–709. 10.1007/s13311-019-00715-6 30945124 PMC6694355

[B13] Huang Y. Wang Y. Wang H. Liu Z. Yu X. Yan J. (2019). Prevalence of mental disorders in China: a cross-sectional epidemiological study. *Lancet Psychiatry* 6 211–224. 10.1016/S2215-0366(18)30511-X 30792114

[B14] Huang S. W. Wang W. T. Chou L. C. Liao C. D. Liou T. H. Lin H. W. (2015). Osteoarthritis increases the risk of dementia: a nationwide cohort study in Taiwan. *Sci. Rep.* 5:10145. 10.1038/srep10145 25984812 PMC4434986

[B15] Innes K. E. Sambamoorthi U. (2020). The association of osteoarthritis and related pain burden to incident alzheimer’s disease and related dementias: a retrospective cohort study of U.S. medicare beneficiaries. *J. Alzheimers Dis.* 75 789–805. 10.3233/JAD-191311 32333589 PMC7418050

[B16] Katchamart W. Narongroeknawin P. Phutthinart N. Srinonprasert V. Muangpaisan W. Chaiamnauy S. (2019). Disease activity is associated with cognitive impairment in patients with rheumatoid arthritis. *Clin. Rheumatol.* 38 1851–1856. 10.1007/s10067-019-04488-3 30848400

[B17] Li C. Liu T. Sun W. Wu L. Zou Z. Y. (2015). Prevalence and risk factors of arthritis in a middle-aged and older Chinese population: the China health and retirement longitudinal study. *Rheumatology (Oxford)* 54 697–706. 10.1093/rheumatology/keu391 25288780

[B18] Lin T. M. Chen W. S. Sheu J. J. Chen Y. H. Chen J. H. Chang C. C. (2018). Autoimmune rheumatic diseases increase dementia risk in middle-aged patients: a nationwide cohort study. *PLoS One* 13:e0186475. 10.1371/journal.pone.0186475 29304089 PMC5755737

[B19] Liu L. (2011). 2010 Chinese guidelines for the management of hypertension. *Chin. J. Hypertens.* 39 579–615. 22088239

[B20] Meade T. Manolios N. Cumming S. R. Conaghan P. G. Katz P. (2018). Cognitive impairment in rheumatoid arthritis: a systematic review. *Arthritis Care Res. (Hoboken)* 70 39–52. 10.1002/acr.23243 28371512

[B21] Min C. Bang W. J. Kim M. Oh D. J. Choi H. G. (2020). Rheumatoid arthritis and neurodegenerative dementia: a nested case-control study and a follow-up study using a national sample cohort. *Clin. Rheumatol.* 39 159–166. 10.1007/s10067-019-04769-x 31523786

[B22] Newby D. Prieto-Alhambra D. Duarte-Salles T. Ansell D. Pedersen L. van der Lei J. (2020). Methotrexate and relative risk of dementia amongst patients with rheumatoid arthritis: a multi-national multi-database case-control study. *Alzheimers Res. Ther.* 12:38. 10.1186/s13195-020-00606-5 32252806 PMC7137292

[B23] Pan X. F. Wang L. Pan A. (2021). Epidemiology and determinants of obesity in China. *Lancet Diabetes Endocrinol.* 9 373–392. 10.1016/s2213-8587(21)00045-0 34022156

[B24] Petersen L. E. Baptista T. S. A. Molina J. K. Motta J. G. do Prado A. Piovesan D. M. (2018). Cognitive impairment in rheumatoid arthritis: role of lymphocyte subsets, cytokines and neurotrophic factors. *Clin. Rheumatol.* 37 1171–1181. 10.1007/s10067-018-3990-9 29372349

[B25] Petersen R. C. Lopez O. Armstrong M. J. Getchius T. S. D. Ganguli M. Gloss D. (2018). Practice guideline update summary: mild cognitive impairment: report of the guideline development, dissemination, and implementation subcommittee of the American Academy of Neurology. *Neurology* 90 126–135. 10.1212/WNL.0000000000004826 29282327 PMC5772157

[B26] Policicchio S. Ahmad A. N. Powell J. F. Proitsi P. (2017). Rheumatoid arthritis and risk for Alzheimer’s disease: a systematic review and meta-analysis and a Mendelian Randomization study. *Sci. Rep.* 7:12861. 10.1038/s41598-017-13168-8 28993680 PMC5634412

[B27] Sha T. Cheng W. Yan Y. (2018). Prospective associations between pulse pressure and cognitive performance in Chinese middle-aged and older population across a 5-year study period. *Alzheimer’s Res. Ther.* 10:29. 10.1186/s13195-018-0355-1 29530075 PMC5848624

[B28] Sturgeon J. A. Finan P. H. Zautra A. J. (2016). Affective disturbance in rheumatoid arthritis: psychological and disease-related pathways. *Nat. Rev. Rheumatol.* 12 532–542. 27411910 10.1038/nrrheum.2016.112 PMC5449457

[B29] Ungprasert P. Wijarnpreecha K. Thongprayoon C. (2016). Rheumatoid arthritis and the risk of dementia: a systematic review and meta-analysis. *Neurol. India* 64 56–61. 10.4103/0028-3886.173623 26754993

[B30] Veeranki S. P. Downer B. Jupiter D. Wong R. (2017). Arthritis and risk of cognitive and functional impairment in older Mexican adults. *J. Aging Health* 29 454–473. 10.1177/0898264316636838 26965081 PMC5017899

[B31] Wallin K. Solomon A. Kåreholt I. Tuomilehto J. Soininen H. Kivipelto M. (2012). Midlife rheumatoid arthritis increases the risk of cognitive impairment two decades later: a population-based study. *J. Alzheimers Dis.* 31 669–676. 10.3233/jad-2012-111736 22647255

[B32] Xue J. Li J. Liang J. Chen S. (2018). The prevalence of mild cognitive impairment in China: a systematic review. *Aging Dis.* 9 706–715. 10.14336/AD.2017.0928 30090658 PMC6065290

[B33] Xue Q. Pan A. Gong J. Wen Y. Peng X. Pan J. (2020). Association between arthritis and depression risk: a prospective study and meta-analysis. *J. Affect. Disord.* 273 493–499. 10.1016/j.jad.2020.04.038 32560945

[B34] Yang X. Pan A. Gong J. Wen Y. Ye Y. Wu J. H. (2020). Prospective associations between depressive symptoms and cognitive functions in middle-aged and elderly Chinese adults. *J. Affect. Disord.* 263 692–697. 10.1016/j.jad.2019.11.048 31744738

[B35] Zhao Y. Hu Y. Smith J. P. Strauss J. Yang G. (2014). Cohort profile: the China Health and Retirement Longitudinal Study (CHARLS). *Int. J. Epidemiol.* 43 61–68. 10.1093/ije/dys203 23243115 PMC3937970

