# Supplementary material for: Prospective Evaluation of the Association Between Arthritis and Cognitive Functions in Middle-Aged and Elderly Chinese
Source: Front Aging Neurosci. 2021 Oct 27;13:687780. doi: 10.3389/fnagi.2021.687780 (PMC8579809; doi:10.3389/fnagi.2021.687780)
Supplement: Supplementary file 1 [file Data_Sheet_1.docx]

**List of supplementary tables and figures**

Supplementary Table 1 Comparison of baseline characteristics between participants included and excluded

Supplementary Table 2 Associations between baseline arthritis and subsequent cognitive functions after multiple imputation (*n* = 8,996)

Supplementary Figure 1 Flowchart of participant eligibility in the China Health and Retirement Longitudinal Study

Supplementary Table 1. Comparison of baseline characteristics between participants included and excluded

| Characteristics | Total (%) | Arthritis | | *P* values |
| --- | --- | --- | --- | --- |
|  |  | Included | Excluded |  |
| Total | 8,996 (100.00) | 7,529 (83.69) | 1,467 (16.31) |  |
| Age, mean (SD), years | 57.45 (8.94) | 57.53 (8.85) | 57.07 (9.36) | 0.072 |
| BMI, mean (SD), kg/m^2^ | 23.81 (3.73) | 23.81 (3.71) | 24.09 (4.75) | 0.427 |
| Sex, n (%) |  |  |  | 0.487 |
| Male | 4,490 (49.96) | 3,749 (49.79) | 741 (50.79) |  |
| Female | 4,498 (50.04) | 3,780 (50.21) | 718 (49.21) |  |
| Residence, n (%) |  |  |  | <0.001 |
| Rural | 6,602 (73.45) | 5,744 (76.29) | 858 (58.81) |  |
| Urban | 2,386 (26.55) | 1,785 (23.71) | 601 (41.19) |  |
| Marital status, n (%) |  |  |  | 0.400 |
| Married | 8,937 (99.34) | 7,482 (99.38) | 1,455 (99.18) |  |
| Unmarried | 59 (0.66) | 47 (0.62) | 12 (0.82) |  |
| Education level, n (%) |  |  |  | <0.001 |
| Illiterate | 1,521 (16.91) | 1,325 (17.60) | 196 (13.39) |  |
| Primary/middle school | 3,724 (41.41) | 3,198 (42.48) | 526 (35.93) |  |
| High school or above | 3,748 (41.68) | 3,006 (39.93) | 742 (50.68) |  |
| Smoking status, n (%) |  |  |  | 0.264 |
| Never smoking | 5,378 (59.79) | 4,476 (59.45) | 902 (61.53) |  |
| Former smoking | 800 (8.89) | 669 (8.89) | 131 (8.94) |  |
| Current smoking | 2,817 (31.32) | 2,384 (31.66) | 433 (29.54) |  |
| Drinking status, n (%) | |  |  | 0.834 |
| Never drinking | 6,095 (67.75) | 5,105 (67.80) | 5,105 (67.80) |  |
| former drinking | 512 (5.96) | 432 (5.74) | 432 (5.74) |  |
| current drinking | 2,389 (26.56) | 1,992 (26.46) | 1,992 (26.46) |  |
| Comorbidities, n (%) |  |  |  | <0.001 |
| None | 3,516 (44.04) | 3,473 (46.13) | 43 (9.47) |  |
| One | 2,984 (37.38) | 2,780 (36.92) | 204 (44.93) |  |
| Two | 1,144 (14.33) | 988 (13.12) | 156 (34.36) |  |
| Three or more | 339 (4.25) | 288 (3.83) | 51 (11.23) |  |
| Arthritis, n (%) | 2,875 (31.96) | 2,426 (32.22) | 449 (30.61) | 0.225 |
| Yes | 2,875 (31.96) | 2,426 (32.22) | 449 (30.61) |  |
| No | 6,121 (68.04) | 5,103 (67.78) | 1,018 (69.39) |  |

Abbreviations: BMI: body mass index; SD, standard deviation.

Supplementary Table 2. Associations between baseline arthritis and subsequent cognitive functions after multiple imputation (*n* = 8,996)

|  | Model 1: *β* (95% CI) ^a^ | Model 2: *β* (95% CI) ^a^ | Model 3: *β* (95% CI) ^a^ |
| --- | --- | --- | --- |
| Episodic memory | -0.25 (-0.29, -0.20) | -0.09 (-0.13, -0.05) | -0.08 (-0.12, -0.04) |
| Mental status | -0.48 (-0.55, -0.41) | -0.17 (-0.23, -0.11) | -0.17 (-0.23, -0.10) |
| Global cognition | -0.73 (-0.83, -0.64) | -0.26 (-0.35, -0.18) | -0.25 (-0.34, -0.17) |

Abbreviations: CI, confidence interval

Model 1: adjusted for years since baseline (0, 2, and 4 years).

Model 2: adjusted for age (continuous, years), sex (male and female), marital status (married and unmarried), education (illiterate, primary/middle school, and senior high school or above), residential area (rural and urban), BMI (continuous, kg/m^2^), smoking status (never, former, and current), drinking status (never, former, and current), and variables in Model 1.

Model 3: adjusted for comorbidities (none, one, two, and three or more) and variables in Model 2.

^a^ For arthritis versus no arthritis.

Supplementary Figure 1 Flowchart of participant eligibility in the China Health and Retirement Longitudinal Study

The China Health and Retirement Longitudinal Study

Baseline survey

(2011-2012; *n* = 17,708)

Included for analysis (*n* = 7,529)

Participants with data missing for arthritis at baseline (*n* = 31)

Participants with data missing for cognitive functions at baseline or at both follow-ups (*n* = 8,191)

Participants with memory-related disease (Alzheimer’s disease, brain atrophy, and Parkinson’s disease) or missing data of memory-related disease at baseline (*n* = 490)

Participants with data missing for major sociodemographics, behaviors and lifestyle covariates (*n* = 1,467) including sex (*n* = 8), residential area (*n* = 8), education (*n* = 3), smoking status (*n* = 1), BMI (*n* = 1,347), and comorbidities (*n* = 100).
